# The existence of solutions of *q*-difference-differential equations

**DOI:** 10.1186/s40064-016-2179-4

**Published:** 2016-05-04

**Authors:** Xin-Li Wang, Hua Wang, Hong-Yan Xu

**Affiliations:** College of Science, University of Shanghai for Science and Technology, Shanghai, 200093 People’s Republic of China; Department of Informatics and Engineering, Jingdezhen Ceramic Institute, Jingdezhen, 333403 Jiangxi People’s Republic of China

**Keywords:** Transcendental, *q*-Difference differential equation, Solution, Zero order, 39A 50, 30D 35

## Abstract

By using the Nevanlinna theory of value distribution, we investigate the existence of solutions of some types of non-linear *q*-difference differential equations. In particular, we generalize the Rellich–Wittich-type theorem and Malmquist-type theorem about differential equations to the case of *q*-difference differential equations (system).

## Background

In this paper, we shall assume that readers are familiar with the basic theorems and the standard notations of the Nevanlinna value distribution theory of meromorphic functions such as *m*(*r*, *f*), $N(r,f), T(r,f), \ldots$, (see Hayman 1964; Yang 1993; Yi and Yang 1995). For a meromorphic function *f*, *S*(*r*, *f*) denotes any quantity satisfying $S(r,f)=o(T(r,f))$ for all *r* outside a possible exceptional set of finite logarithmic measure, $\mathbb {S}(f)$ denotes the family of all meromorphic function *a*(*z*) such that $T(r,a)=S(r,f)=o(T(r,f))$, where $r\rightarrow \infty$ outside of a possible exceptional set of finite logarithmic measure. In addition, we denote by $S_1(r,f)$ any quantity satisfying $S_1(r,f)=o(T(r,f))$ for all *r* on a set *F* of logarithmic density 1, the logarithmic density of a set *F* is defined by

$$\limsup _{r\rightarrow \infty }\frac{1}{\log r}\int _{[1,r]\cap F}\frac{1}{t}dt.$$

Throughout this paper, the set *F* of logarithmic density can be not necessarily the same at each occurrence.

Complex differential equations have attracted many mathematicians, and there are many results about the existence or growth of solutions of differential equations (see He 1981; Laine 1993, 1971; Liao 2015; Tu et al. 2013). In recent, with the development of Nevanlinna theory in complex difference equations (see Barnett et al. 2007; Chiang and Feng 2008; Gundersen et al. 2002; Halburd and Korhonen 2006a, b), there has been an increasing interest in studying difference equations, difference product and *q*-difference in the complex plane $\mathbb {C}$, a number of papers (including Chen 2010; Gan 2015; Halburd and Korhonen 2007; Heittokangas et al. 2001; Laine and Yang 2007; Qi and Yang 2015; Zheng and Chen 2010; Zhang and Korhonen 2010) have focused on the existence and growth of solutions of difference equation.

The following two results had been proved by F. Rellich and H. Wittich, respectively.

### **Theorem 1**

(see He 1981, Rellich). *Let the differential equation be the following form*

1 $$w'(z) = f(w),$$

*Iff*(*w*) *is transcendental meromorphic function ofw*, *then Eq.* (1) *has no non-constant entire solution.*

Wittich (1955) studied the more general differential equation than Eq. (1) and obtained the following result.

### **Theorem 2**

(see Wittich 1955). *Let*

$$\Phi (z,w)=\sum a_{(i)}(z)w^{i_0}(w')^{i_1}\cdots (w^{(n)})^{i_n}$$

*be differential polynomial, with coefficients*$a_{(i)}(z)$*are polynomial ofz*. *If the right-hand side of the differential equation*

2 $$\Phi (z,w)=f(w),$$

*f(w)is the transcendental meromorphic function ofw, then the Eq. (*2*) has no non-constant entire solution.*

In the 1980s, Yanagihara and Shimomura extended the above type theorem to the case of difference equations (see Yanagihara 1980, 1983; Shimomura 1981), and obtained the following two results

### **Theorem 3**

(see Shimomura 1981). *For any non-constant polynomialP*(*w*), *the difference equation*

$$w(z + 1) = P(w(z))$$

*has a non-trivial entire solution.*

### **Theorem 4**

(see Yanagihara 1980). *For any non-constant rational functionR(w),the difference equation*

$$\begin{aligned} w(z + 1) = R(w(z)) \end{aligned}$$

*has a non-trivial meromorphic solution in the complex plane.*

## Conclusions and our main results

In the present paper, we mainly study the above Rellich–Wittich-type theorem of *q*-difference differential equation (system).

### **Definition 5**

We call the equation a *q*-difference differential equation (system) if a equation (system) contains the *q*-difference term *f*(*qz*) and differential term $f'(z)$ of one function *f*(*z*) at the same time.

We consider the system of *q*-difference differential equation of the form

3 $$\begin{aligned} \left\{ \begin{aligned} \Omega _1 (z,w_1):=\sum _{J_1}a_{J_1}(z)\prod _{j=1}^{n_1}(w_1^{(j)}(q_jz))^{i_j}=P_s[f(w_2)],\\ \Omega _2(z,w_2):=\sum _{J_2}b_{J_2}(z)\prod _{j=1}^{n_2}(w_2^{(j)}(q_jz))^{i_j}=P_t[f(w_1)], \end{aligned} \right. \end{aligned}$$

where $a_{J_1}(z),b_{J_2}(z)$ are polynomials of *z* and $q\in \mathbb {C}{\setminus }\{0\}$, $P_m[f]$ is a polynomial of *f* of degree *m*,

$$P_m[f]=d_m(z)f^m+d_{m-1}(z)f^{m-1}+\cdots +d_0(z),$$

and $d_m(z),\ldots ,d_0(z)$ are polynomials of *z*, and obtain the following results.

### **Theorem 6**

*For system* (3), *if*$s\ge 1, t\ge 1$ and *fis a transcendental meromorphic function, then the system* (3) *has no non-constant transcendental entire solutions*$(w_1,w_2)$*with zero order.*

### *Remark 7*

Under the assumptions of Theorem 6, the system of *q*-difference differential equation

$$\begin{aligned}\left\{ \begin{aligned} \sum _{J_1}a_{J_1}(z)\prod _{j=1}^{n_1}(w_1^{(j)}(q_jz))^{i_j}=\frac{P_{s_2}[f(w_2)]}{Q_{t_2}[f(w_2)]},\\ \sum _{J_2}b_{J_2}(z)\prod _{j=1}^{n_2}(w_2^{(j)}(q_jz))^{i_j}=\frac{P_{s_1}[f(w_1)]}{Q_{t_1}[f(w_1)]}, \end{aligned} \right. \end{aligned}$$

has no non-constant transcendental entire solutions $(w_1,w_2)$ with zero order, where $s_1,s_2\ge 1$ and $P_{s_i}[f]$ and $Q_{t_i}[f]$ are irreducible polynomials in *f*.

If $s=t$ and $w_1=w_2$, we can get the following theorem easily

### **Theorem 8**

*Let*

4 $$\begin{aligned} \Omega (z,w):=\sum _{J}a_{J}(z)\prod _{j=1}^n\left( w^{(j)}(q_jz)^{i_j}\right) =P_s[f(w)], \end{aligned}$$

*if*$s\ge 1$*andfis a transcendental meromorphic function, thenthe system* (4) *has no non-constant transcendental entiresolution with zero order.*

From Remark 7, we have

### *Remark 9*

Let $s\ge 1$ and *f* be a transcendental meromorphic function, then the equation

$$\sum _{J}a_{J}(z)\prod _{j=1}^n\left( w^{(j)}(q_jz)^{i_j}\right) =\frac{P_{s}[f(w)]}{Q_{t}[f(w)]},$$

has no non-constant transcendental entire solution with zero order, where $P_{s}[f]$ and $Q_{t}[f]$ are irreducible polynomials in *f*.

As we know, it is very interest problem about the Malmquist theorem of differential equations, Laine (1993) gave the following results

### **Theorem 9**

(see Laine 1993). *Let*

5 $$(w'(z))^n = R(z,w),$$

*whereR*(*z*, *w*) *is defined as*

$$R(z,w)=\frac{\sum _{i=0}^ka_i(z)}{\sum _{j=0}^lb_j(z)}.$$

*If Eq.* (5) *has transcendental meromorphic solution, then there will be*$l= 0$*and*$k\le 2n$.

### **Theorem 10**

(see Laine 1993). *Let*

6 $$\sum a_{(i)}(z)w^{i_0}(w')^{i_1}\cdots (w^{(n)})^{i_n}= R(z,w),$$

*whereR*(*z*, *w*) *is defined as in Theorem* 9. *If Eq.* (6) *has transcendental meromorphic solution, then there will be*$l= 0$*and*$k\le \min \{\Delta ,\lambda +\mu (1- \Theta (\infty ))\}$, *where*

$$\triangle =\max \left\{ \sum _{\alpha =0}^n(1+\alpha )i_\alpha \right\} ,~~ \lambda =\max \left\{ \sum _{\alpha =0}^ni_\alpha \right\} ,$$

*and*

$$\mu =\max \left\{ \sum _{\alpha =0}^n\alpha i_\alpha \right\} ,~~~\Theta (\infty )=1-\limsup _{r\rightarrow \infty }\frac{\overline{N}(r,w)}{T(r,w)}.$$

Recently, there were a number of papers focused on the Malmquist-type theorem of the complex difference equations. Ablowitz et al. (2000) proved some results on the classical Malmquist-type theorem of the complex difference equations by applying Nevanlinna theory. Besides, Gao, Xu and Li also studied some systems of complex difference equation and obtained some more precise results related to Malmquist-type theorem (see Gao 2012a, b, c; Li and Gao 2015; Xu et al. 2013, 2015; Xu and Xuan 2015). In this paper, we mainly study the *q*-difference differential equation about the Maimquist-type theorem, and obtain the following theorem.

### **Theorem 11**

*Let*

7 $$(w'(qz))^n = R(z,w),$$

*whereR*(*z*, *w*) *is defined as*

$$R(z,w)=\frac{P(z,w)}{Q(z,w)}=\frac{\sum _{i=0}^ka_i(z)w^i}{\sum _{j=0}^lb_j(z)w^j},$$

*P*(*z*, *w*) *andQ*(*z*, *w*) *are irreducible polynomials inw*, *coefficients*$a_i(z),b_j(z)$*are rational functions ofz*. *IfEq.* (7) *exists transcendental meromorphic solutionswith zero order, then we also think that*$l= 0$*and*$k\le 2n$.

Similar to the proof of Theorem 11, we can get the following corollary easily.

### **Corollary 12**

*Let*

8 $$\begin{aligned} \sum a_{(i)}(z)w^{i_0}(w')^{i_1}(q_1z)\cdots (w^{(n)}(q_nz))^{i_n}= R(z,w), \end{aligned}$$

*whereR*(*z*, *w*) *is defined as in Theorem* 11. *If Eq.* (8) *has transcendental meromorphic solution of zero order*, *then there will be*$l= 0$*and*$k\le \min \{\Delta ,\lambda +\mu (1- \Theta (\infty ))\}$, *where*$\Delta , \lambda$*and*$\mu$*are stated as in Theorem* 10.

## Some Lemmas

### **Lemma 13**

(Valiron-Mohon’ko, Laine 1993). Let *f*(*z*) *be a meromorphic function. Then for all irreducible rational functions inf*,

$$R(z,f(z))=\frac{\sum _{i=0}^ma_i(z)f(z)^i}{\sum _{j=0}^nb_j(z)f(z)^j},$$

*with meromorphic coefficients*$a_i(z),b_j(z)$, *the characteristic function ofR*(*z*, *f*(*z*)) *satisfies*

$$T(r,R(z,f(z)))=dT(r,f)+O(\Psi (r)),$$

*where*$$d=\max \{m,n\}$$*and*$$\Psi (r)=\max _{i,j}\{T(r,a_i),T(r,b_j)\}$$.

### **Lemma 14**

(Zhang and Korhonen 2010, Theorem 1 and Theorem 3) *Letf*(*z*) *be a transcendental meromorphic function of zero order and q be a nonzero complex constant. Then*

$$\begin{aligned} T(r,f(qz)) = (1+o(1))T(r, f(z)) \end{aligned}$$

*and*

$$\begin{aligned} N(r, f(qz)) = (1+o(1))N(r, f(z)), \end{aligned}$$

*on a set of logarithmic density 1*.

### **Lemma 15**

(see Barnett et al. 2007). *Letf*(*z*) *be a nonconstant zero-order meromorphic function and*$q \in \mathbb {C}{\setminus } \{0\}$. *Then*

$$m\left( r,\frac{f(qz)}{f(z)}\right) =S(r,f),$$

*on a set of logarithmic density 1 for all r outside a possible exceptional set of logarithmic density 0.*

### **Lemma 16**

(see Yi and Yang 1995, p. 37 or Yang 1993). *Letf*(*z*) *be a nonconstant meromorphic function in the complex plane andlbe a positive integer. Then*

$$N(r,f^{(l)})= N(r,f)+l\overline{N}(r,f), ~~T(r,f^{(l)})\le T(r,f)+l\overline{N}(r,f)+S(r,f).$$

### **Lemma 17**

*Let*$q\in \mathbb {C}{\setminus }\{0\}$*andf*(*z*) *be a nonconstant meromorphic function with zero order. Then for any positive finite integerk*, *we have*

$$m\left( r,\frac{f^{(k)}(qz)}{f(z)}\right) =S_1(r,f),$$

*and*

$$m\left( r,f^{(k)}(qz)\right) \le m(r,f)+S_1(r,f).$$

### *Proof*

It follows from Lemma 15 that

$$m\left( r,\frac{f^{(k)}(qz)}{f(z)}\right) \le m\left( r,\frac{f^{(k)}(qz)}{f(qz)}\right) +m\left( r,\frac{f(qz)}{f(z)}\right) =S_1(r,f).$$

Moreover, we have

$$m\left( r,f^{(k)}(qz)\right) = m\left( r,\frac{f^{(k)}(qz)}{f(z)}f(z)\right) \le m(r,f)+S_1(r,f).$$

This completes the proof of Lemma 17.$\square$

## The Proof of Theorem 6

Suppose that $w_i (i=1,2)$ be non-constant entire functions solutions of system (3) with zero order. Suppose $i=1$, let $E_1=\{z: |w_1(z)|>1\}$ and $E_2=\{z: |w_1(z)|\le 1\}$, then we have

$$\begin{aligned} |\Omega _1(z,w_1)|&=\left| \sum _{J_1}a_{J_1}(z)(w_1(z))^{\lambda _i} \left( \frac{w_1'(q_1z)}{w_1(z)}\right) ^{i_1}\cdots \left( \frac{w_1'(q_{n_1}z)}{w_1(z)}\right) ^{i_{n_1}}\right| \\&\le \left\{ \begin{array} {ll} |w_1(z)|^\lambda \sum _{J_1}|a_{J_1}(z)|\left| \frac{w_1'(q_1z)}{w_1(z)}\right| ^{i_1}\cdots \left| \frac{w_1'(q_{n_1}z)}{w_1(z)}\right| ^{i_{n_1}},~&{}~\ if ~~z\in E_1,\\ \sum _{J_1}|a_{J_1}(z)|\left| \frac{w_1'(q_1z)}{w_1(z)}\right| ^{i_1}\cdots \left| \frac{w_1'(q_{n_1}z)}{w_1(z)}\right| ^{i_{n_1}},~~&{}\ if ~~z\in E_2, \end{array}\right. \end{aligned}$$

where $\lambda =\max \{\lambda _i\}, \lambda _i=i_1+\cdots +i_{n_1}$. It follows from Lemma 15 and 17 that

$$m(r,\Omega _1(z,w_1))=\frac{1}{2\pi }\left( \int _{E_1}+\int _{E_2}\right) \log ^+|\Omega _1(z,w_1)|d\theta \le \lambda m(r,w_1)+S_1(r,w_1).$$

And since $w_1(z)$ is a non-constant entire function, we have $N(r,w_1)=0$. Thus, we have $N(r, \Omega _1(z,w_1)=0$ and

9 $$T(r,\Omega _1)=m(r,\Omega _1)\le \lambda m(r,w_1)+S_1(r,w_1).$$

Similarly, we have

10 $$T(r,\Omega _2)=m(r,\Omega _2)\le \eta m(r,w_2)+S_1(r,w_2),$$

where $$\eta =\max \{\eta _i\}, \eta _i=i_1+\cdots +i_{n_2}$$.

Since $P_s[f(w_2)]$ is a polynomial of $f(w_2)$, we can take a complex constant $\alpha$ such that

$$P_s[f(w_2)]-\alpha =[f(w_2)-\beta _1]\cdots [f(w_2)-\beta _s],$$

where $$\beta _1, \ldots ,\beta _s$$ are complex constants, and there at least exists a constant $$\beta \in \{\beta _1, \ldots ,$$$$\beta _s\}$$ which is not Picard exceptional value of $$f(w_2)$$. Let $$\{\xi _j, j=1,2,\ldots , p_2\}$$ be the zeros of $$f(w_2)-\beta$$, where $$p_2$$ is any positive integer with $$p_2\ge 1$$. Then it follows

11 $$\sum _{j=1}^{p_2}N\left(r,\frac{1}{w_2-\xi _j}\right)\le N\left(r,\frac{1}{f(w_2)-\beta }\right) \le N\left(r,\frac{1}{P_s[f(w_2)]-\alpha }\right).$$

Thus, by using the second main theorem and (10), (11), we can get that

12 $$\begin{aligned} (p_2-2)T(r,w_2)&\le \sum _{j=1}^{p_2}N\left(r,\frac{1}{w_2-\xi _j}\right)+S(r,w_2)\\ \nonumber&\le N\left(r,\frac{1}{P_s[f(w_2)]-\alpha }\right)+S(r,w_2) \\ \nonumber&\le T(r,P_s[f(w_2)])+S(r,w_2)\\ \nonumber&\le T(r,\Omega _1(z,w_1))+S(r,w_2)\\&\le \lambda T(r,w_1)+S_1(r,w_1)+S_1(r,w_2). \end{aligned}$$

Similarly, there exists any positive integer $p_1(\ge 1)$ such that

13 $$(p_1-2)T(r,w_1)\le \eta T(r,w_2)+S_1(r,w_1)+S_1(r,w_2).$$

It follows from (12) and (13) that

14 $$\left[ (p_1-2)(p_2-2)-\lambda \eta \right] T(r,w_i)\le S_1(r,w_1)+S_1(r,w_2),$$

Since $w_i (i=1,2)$ are transcendental and $p_1,p_2$ are arbitrary, we can get a contradiction with (4). Hence, we complete the proof of Theorem 6.

## The Proof of Theorem 11

We firstly choose a constant $a\in \mathbb {C}$ such that $P(z, a)\ne 0$ and $Q(z,a)\ne 0$, then (7) can be rewritten as

15 $$(w'(qz))^n=\frac{P(z,a)+A_1(z)(w-a)+\cdots +A_k(z)(w-a)^k}{Q(z,a)+B_1(z)(w-a)+\cdots +B_l(z)(w-a)^l},$$

where $A_1(z), \ldots ,A_k(z), B_1(z),\ldots ,B_l(z)$ are all rational functions. Let $\varphi (z)=\frac{1}{w(z)-a}$, that is, $w(z)=\frac{1}{\varphi (z)}+a$ and

16 $$(w'(qz))^n=(-1)^n\varphi (qz)^{-2n}(\varphi '(qz))^n.$$

Hence, it follows from (15) and (16) that

17 $$\begin{aligned} \nonumber (\varphi '(qz))^n&= (-1)^{-n}\varphi (qz)^{2n}(w'(qz))^n\\ \nonumber&=(-1)^{-n}\varphi (qz)^{2n} \frac{P(z,a)+A_1(z)\varphi (z)^{-1}+\cdots +A_k(z)\varphi (z)^{-k}}{Q(z,a)+B_1(z)\varphi (z)^{-1}+\cdots +B_l(z)\varphi (z)^{-l}} \\ \nonumber&=: \varphi (qz)^{2n}\varphi (z)^{l-k} \frac{\sum _{i=0}^k\widetilde{a}_i(z)\varphi (z)^i}{\sum _{i=0}^l\widetilde{b}_i(z)\varphi (z)^i}\\&=\frac{\widetilde{P}(z,\varphi (z))}{\widetilde{Q}(z,\varphi (z))}=\widetilde{R}(z,\varphi (z)), \end{aligned}$$

where $$\widetilde{a}_i(z)=(-1)^{-n}P(z,a)\ne 0, \widetilde{b}_i(z)=Q(z,a)\ne 0$$.

Suppose that *w*(*z*) is a transcendental meromorphic solution of equation (7) with zero order, then $\varphi (z)= \frac{1}{w(z)-a}$ is also a transcendental meromorphic solution of Eq. (17). We will discuss two cases as follows.

If $2n+l-k\le 0$, then $\hbox {deg}_\varphi \widetilde{P}(z,\varphi )=k$ and $\hbox {deg}_\varphi \widetilde{Q}(z,\varphi )=l-(2n+l-k)=k-2n$. It follows by Lemma 13 that

$$T(r,\widetilde{R}(z,\varphi (z))=k T(r,\varphi )+S(r,\varphi ).$$

And by Lemmas 13–17, we have

$$\begin{aligned} T(r,(\varphi '(qz))^n)&=nT(r,\varphi '(qz))\\&\le nN(r,\varphi (qz))+n\overline{N}(r,\varphi '(qz))+nm(r,\varphi '(qz))\\&\le 2n T(r,\varphi (qz))+S(r,\varphi )\\&\le 2nT(r,\varphi )+S_1(r,\varphi ). \end{aligned}$$

Thus, it follows

18 $$kT(r,\varphi )\le 2n T(r,\varphi )+S_1(r,\varphi ),$$

which implies $k\le 2n$. Since $2n+l-k\le 0$ and $l\ge 0$, then we have $l=0$.

If $2n+l-k\ge 0$, then then $\hbox {deg}_\varphi \widetilde{P}(z,\varphi )=2n+l$ and $\hbox {deg}_\varphi \widetilde{Q}(z,\varphi )=l$. It follows by Lemma 13 that

$$T(r,\widetilde{R}(z,\varphi (z))=(2n+l)T(r,\varphi )+S(r,\varphi ).$$

Similar to the argument as in above, we can get $l=0$ and $k\le 2n$.

This completes the proof of Theorem 11.
